# Left-sided approach video-assisted thymectomy for the treatment of thymic diseases

**DOI:** 10.1186/1477-7819-12-398

**Published:** 2014-12-29

**Authors:** Yun Li, Jun Wang

**Affiliations:** Department of Thoracic Surgery, People’s Hospital of Peking University, Number 11, Xizhimen South Street, Xicheng District, Beijing, 100044 China

**Keywords:** Left-sided approach, Video-assisted thoracoscopic, Thymectomy

## Abstract

**Background:**

Video-assisted thoracoscopic thymectomy was developed more than 10 years ago and has become a widely accepted surgical approach. Most published reports regarding this procedure have focused on the right-sided approach. Since left-sided approach chest surgery is the first choice in cases of right pleural adhesion, large left thymus tumors, and tumors in close contact with the great vessels of the left pericardium, we performed thoracoscopic thymectomy using the left-sided approach in 52 cases and summarize herein its technical feasibility, indications, and operative steps.

**Methods:**

Between February 2004 and October 2014, 52 patients (24 men, 28 women, median age: 50 years, ranging from 18 to 85 years), underwent a video-assisted thoracoscopic thymectomy using the left-sided approach. All procedures were performed under general anesthesia with single-lung ventilation. Patients were placed in the right lateral decubitus position and three ports were made. The entire hemithorax was carefully examined, then mediastinal pleura was incised, and the thymus was bluntly dissected from the inferior polar extending to the superior polar. The thymic venous was clipped.

**Results:**

All procedures were carried out safely, including simple thymectomy (n = 43) and extended thymectomy (n = 9). There were no operative deaths or serious complications, and there were seven cases of conversion to open thoracotomy. The mean operative duration was 105.3 minutes (ranging from 80 to 140 minutes). The mean blood loss was 78.5 ml (ranging from 20 to 200 ml), and there was no blood transfusion required. All patients were well throughout the follow-up period.

**Conclusions:**

The left-sided approach for video-assisted thoracoscopic thymectomy was a safe approach and could be an alternative procedure to the right-sided approach for the same procedure.

## Background

Thymic diseases are uncommon lesions which may be asymptomatic or may accompany thoracic complaints or paraneoplastic syndromes. Diagnostic imaging is crucial in order to detect the presence and type of the thymic disease [1, 2]. Thymectomy was the standard method for the treatment of thymic disease. Video-assisted thoracoscopic thymectomy was developed more than 10 years ago and has become a widely accepted surgical approach [3, 4]. Indications for a simple thymectomy are thymic lesions without myasthenia gravis (MG), including small thymoma (Masaoka stage I to II, part of stage III, tumor not touching surrounding tissue) mature teratoma, thymic cyst, thymic hyperplasia, and thymic carcinoma. Accordingly, indications for an extended thymectomy are thymic lesions with MG, including thymoma, mature teratoma, thymic cyst, thymic hyperplasia, and thymic carcinoma [5]. It has become the preferred and standard operation for the treatment of thymic disease. Most published reports regarding this procedure have focused on the right-sided approach, which has been adopted by most surgeons as the space of right chest cavity is relatively large, with little interference from the heart, and with the superior vena cava as anatomical landmark. Few reports focus on the left-sided procedure. Similar to all thoracoscopic surgery, pleural adhesion, tumor volume, and vessel invasion must be taken into consideration. Left-sided approach chest surgery is the first choice in cases of right pleural adhesion, large left thymus tumors, and tumors in close contact with the great vessels of the left pericardium, which are regarded as relative contraindications for the right-sided approach [6, 7]. Thus, we performed thoracoscopic thymectomy using the left-sided approach in 52 cases and summarize herein its technical feasibility, indications, and operative steps.

## Methods

### General data

**Figure 1:**
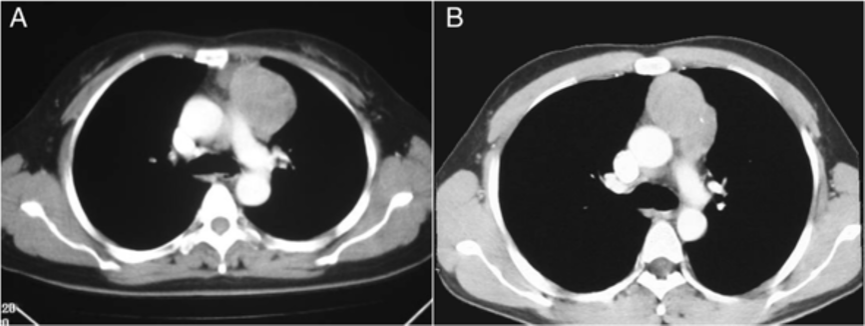
**Computed tomography scans of two patients with huge left side thymomas. A**: A 43-year-old female patient with a solid tumor located at the left side anterior mediastinum, the diameter of the tumor was 5.6 cm; **B**: A 56-year-old male patient with a solid tumor located at the left side anterior mediastinum, the diameter of the tumor was 4.2 cm.

Between February 2004 and October 2014, 286 thoracoscopic thymectomies were performed in Peking University People’s Hospital, Beijing. Inclusion criteria for the left-sided approach were that the patient underwent a complete thoracoscopic thymectomy and the surgical approach was on the left side. Exclusion criteria for the left-sided approach were: the surgical approach was on the right, the patient underwent a trans-sternal thymectomy, or the procedure was associated with any other major surgery. Data were retrospectively collected on all patients. The left-sided approach was used for the following indications: a past history of right-sided chest surgery in one case, right pleural adhesion and blockage in four cases, the tumor was closely connected with the left pericardium or left innominate veins in 10 cases, and large solid tumors mainly lying in the left side of the chest occurred in 37 cases. A total of 234 cases used the right-sided approach, and 52 (24 males (46.2%) and 28 females (53.8%)) used the left-sided approach, which included nine patients with myasthenia symptoms and two patients who underwent a median thoracotomy. All six patients with MG had thymoma or thymic hyperplasia. The maximum diameter of the tumor was 5.7 ± 2.4 mm (ranging from 4 to 10 cm) (Figure 1). There were no patients with symptoms like severe chest pain, superior vena cava syndrome, or hoarseness in both groups. There was no significant difference in age, gender, pulmonary function, and co-morbidities between the two groups. This study was conducted with institutional review board approval (review board approval of People’s Hospital of Peking University 2012–33). Consent from patients was waived because patients were not identified individually.

### Operative steps

**Figure 2:**
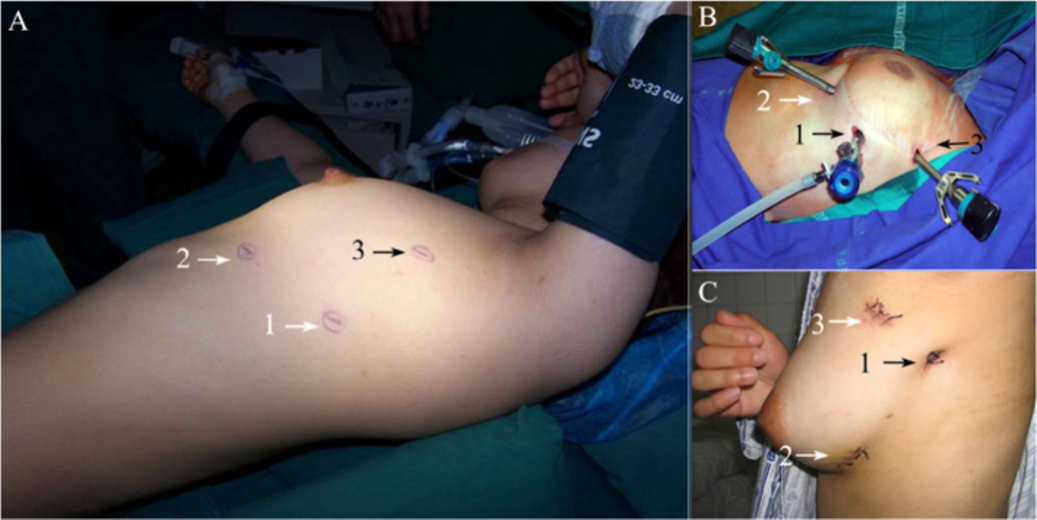
**Schematic diagram for the same patient position and incision location. A**: Patient placed in the right lateral position with retroversion of 30 degrees; **B**: The instrument was placed through the three incisions; **C**: The three incisions were sutured. 1: observation incision was placed at the midaxillary line in the fifth intercostal space; 2: one utility incision was placed at midclavicular line in the fifth intercostal space; 3 the other utility incision was placed at anterior axillary line in the third intercostal space.

**Figure 3:**
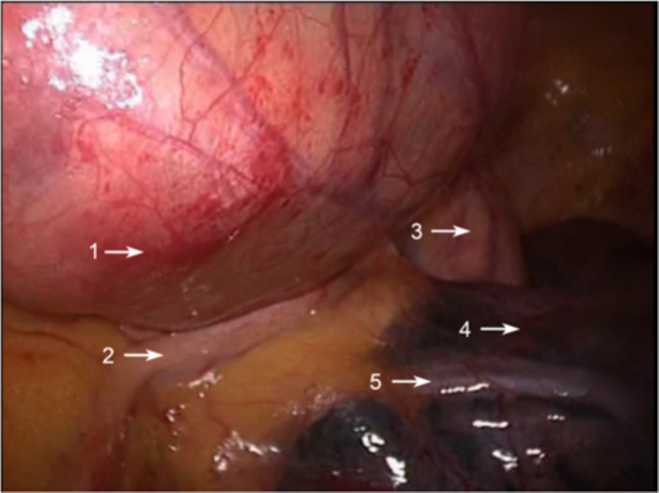
**Intraoperative image of a huge solid thymoma located at the left side anterior mediastinum.** 1: solid thymoma; 2: phrenic nerve of left side; 3: ascending aorta; 4: upper left lobe; 5: pulmonary vein of upper left lobe.

**Figure 4:**
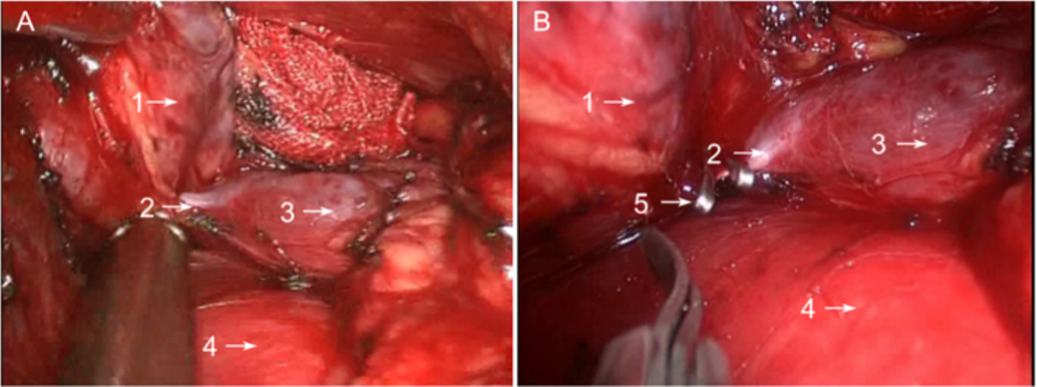
**Exploring and clamping the thymic vein. A**: The thymic vein was explored; **B**: The thymic vein was then clamped with the titanium clip. 1: thymoma; 2: thymic vein; 3: innominate vein of left side; 4: ascending aorta; 5: titanium clip.

All procedures were performed under general anesthesia with double-lumen endotracheal intubation and contralateral one-lung ventilation. The patients were placed in the right lateral position with retroversion of 30 degrees. The operation was performed through three incisions: an observation incision placed at the intersection of the midaxillary line in the fifth intercostal space, and two operating incisions placed at the intersection of the midclavicular line in the fifth intercostal space and at the intersection of anterior axillary line in the third intercostal space, respectively (Figure 2). First, an observation incision was made to put in the thoracoscope and the two operation incisions were made under thoracoscopic guidance. The chest cavity was explored to ensure that the lesions were resectable (Figure 3). Next, the left mediastinal pleural cavity was opened from the lower edge of the internal thoracic artery to the surface of the pericardium, and from the sternum to the anterior margin of the phrenic nerve. The thymus was dissected sharply and/or bluntly by electrocautery or using an ultrasound knife (Johnson, USA) (the usual sequence of dissection: lower left lobe, lower right lobe, upper left lobe, upper right lobe, and isthmus). The bilateral upper gland was stripped down from the neck in order to reveal the thymic vein. Then, the thymic vein was clamped and cut using a titanium clip (Johnson, USA) (Figure 4).

**Figure 5:**
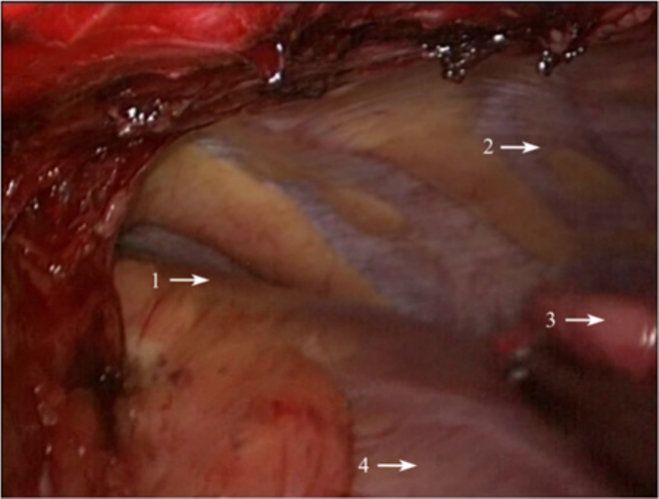
**Schematic diagram for cleansing the right cardiophrenic angle fat tissue via the left-sided approach.** 1: right cardiophrenic angle fat tissue; 2: right chest cavity; 3: lower right lobe; 4: heart.

**Figure 6:**
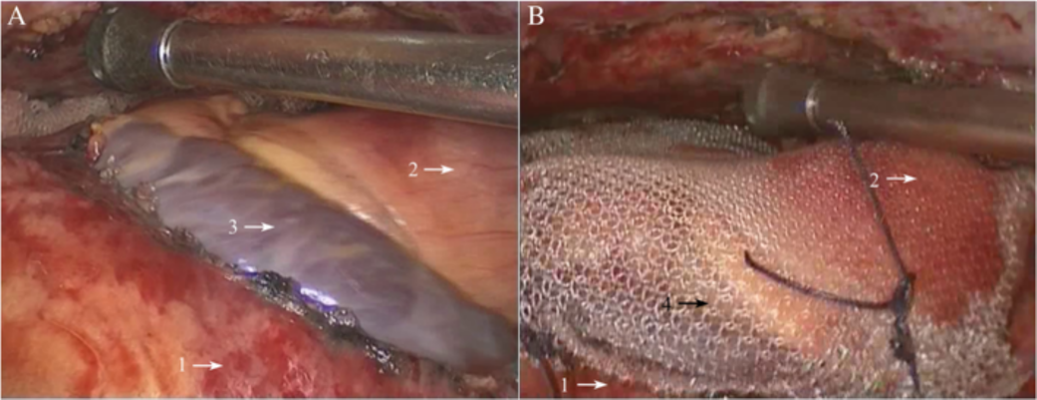
**Repairing the pericardial defects with MARLEX mesh. A**: The defects of the pericardium were explored; **B**: The pericardial defects were repaired with MARLEX mesh. 1: pericardium; 2: ascending aorta; 3: pericardial defects; 4: repairing the pericardial defects with MARLEX mesh.

If necessary, after the removal of thymus, the fatty tissue in the left anterior mediastinum and cardiophrenic angle was cleaned and the right mediastinal pleura was opened to remove the fatty tissue at the right anterior mediastinum and cardiophrenic angle (Figure 5). The removed thymus and fat tissue was placed in a specimen bag to be taken out. A number 28 intrathoracic drain tube (Xudong Jiangsu, China) was maintained in the left side of the chest postoperatively. In the early extensive thymectomy cases that underwent bilateral thoracoscopic surgeries, the thymus and fatty tissue were removed from the left anterior mediastinum and cardiophrenic angle via the left-sided approach, and then the fatty tissue in the right anterior mediastinum and cardiophrenic angle was removed by right-sided thoracoscopic surgery. Occasionally, a thoracotomy was necessary to make a third intercostal incision of about 15 cm without cutting the ribs. Pericardial defects less than 5 cm in diameter were then repaired by continuous cross-stitching under thoracoscope using number 1 absorbable sutures (Covidien USA), whereas for those with a diameter over 5 cm, a MARLEX mesh (Covidien USA) was used to repair the pericardial defects (Figure 6). To repair the innominate vein, the lateral vein walls were continuously sutured using 5–0 nonabsorbable sutures (Covidien USA).

## Results

All procedures were carried out without incident; there was no perioperative death or serious complication. A total of 43 patients received a simple thymectomy, whereas extensive thymectomy was performed in nine patients with MG, including two patients with thymic hyperplasia who underwent bilateral surgery. Whether or not a preoperative biopsy had been obtained did not affect operation decision-making as no preoperative histological diagnosis was obtained. The lesions were located at the upper left lobe of the thymus in six cases, lower left lobe in 44 cases, and evenly distributed hyperplasia occurred in two cases. The postoperative diagnoses of selected cases were as follows: thymoma in 36 cases, thymic cysts in seven patients, thymic carcinoma in six patients, thymic hyperplasia in two patients, and thymic teratoma in one patient. Of the 36 thymomas, 30 patients had Masaoka stage I thymomas, three had stage II thymomas, and three had stage III thymomas. The median operative duration was 105.3 minutes (ranging from 80 to 140 minutes). The operation times in the two bilateral surgery cases were 205 and 210 minutes, respectively. The median intraoperative blood loss was 78.5 ml (ranging from 20 to 200 ml), and no intraoperative or postoperative blood transfusions were administered in this cohort. Tumor invasion of the surrounding organs was observed in 10 cases (19.2%), including seven cases with invasion into the pericardium and three cases with invasion into the left innominate vein. In the seven thoracotomy cases (13.5%), there were three cases where the tumor invaded into the left innominate vein, and so conversion to thoracotomy was performed to repair the lateral wall of the vein. In the other four cases, invasion of the pericardium was observed, and part of the pericardium was resected and repaired using MARLEX mesh. Two patients (3.8%) experienced postoperative complications: one required maintenance of the chest drainage tube for more than seven days and the other experienced a transient cholinergic crisis, which was relieved after mechanical ventilation for two days. The mean follow-up time was 54.3 ± 1.2 months (ranging from 1 to 118 months). No tumor recurrence was observed in all cases and symptoms were significantly improved for all of the nine myasthenia cases.

This group was compared with the 234 cases receiving video-assisted thoracoscopic thymectomy in our hospital using the right-sided approach (Table 1).

**Table 1:** **Comparison of clinical data in thoracoscopic thymectomy between the left and right chest approach**

	Cases	Age (years)	Lesion diameter (cm)	Operative time (min)	Intraoperative blood loss (mL)	Complication rate (%)
Left chest approach	52	47.2 ± 14.5	5.7 ± 2.4	105.3 ± 24.6	78.5 ± 67.6	5.7%
Right chest approach	234	41.3 ± 13.2	4.1 ± 2.9	111.4 ± 48.5	113.2 ± 87.3	4.7%
*P*-value		0.467	0.017	0.251	0.187	

Among the 52 cases treated with the left-sided approach, two cases had diffusive thymic hyperplasia and 50 cases had solid tumors (mainly in the left thymus), including 44 cases in the lower left lobe and six in the upper left lobe. Among the 234 cases treated with the right-sided approach, 37 cases had diffusive thymic hyperplasia and 197 cases had solid tumors, including 149 cases with tumors in the lower right lobe, eight in the isthmus, 10 in the upper right lobe, 28 in the lower left lobe, and two in the upper left lobe. A total of 30 patients with left thymic lesions (including seven with cysts and 23 with thymomas) were treated using the right-sided approach with a maximum average diameter of 2.3 ± 1.4 cm (ranging from 1.2 to 4 cm).

## Discussion

### Operative safety

The right-sided approach is recognized as the standard for thoracoscopic thymectomy. We compared the clinical outcomes between 52 patients who received the left-sided approach and 149 who received the right-sided approach in this study. We found that their operative times, blood losses, and complication rates were similar, and the left-sided approach exhibited satisfactory clinical results, high remission rate, and low recurrence rate, which were consistent with the literature (Table 2), indicating the effectiveness and feasibility of the left-sided approach for thoracoscopic thymectomy.

**Table 2:** **Data on thoracoscopic thymectomy in literature at home and abroad**

	Authors	Year	Cases	Operation time (min)	Blood loss (mL)
Right chest approach	Yasushi [8]	2008	11	163	123
	Torng-Sen Lin [9]	2003	51	150	
	Liu HP et al. [10]	2005	107	90 ± 24	80 ± 22
	Li JF et al. [11]	2013	131	109.1 ± 45.6	101.3 ± 76.5
Left chest approach	Ruckert JC [12]	1999	19	122	100
	Mineo TC [13]	2000	31	148 ± 46	
	This study	2014	52	105.3 ± 23.4	78.5 ± 70.1

### Surgical indications

Mineo *et al*. reported that large thymomas are often located in the left mediastinum, thus all thymectomies should be performed using the left-sided approach [14, 15]. Our study showed that 80 cases had lesions in the left chest, accounting for 28.2% of all tumor cases (diffusive thymic hyperplasia is exempted). Among these 82 cases, 50 cases received left-sided chest surgery, of which 37 cases were due to the fact that the large tumor inclined to the left and the tumor diameter was remarkably larger than those in patients receiving the right-sided approach. Therefore, not all cases are suitable for the left-sided approach. There are several reasons as to why a case may not be suitable for the left-sided approach. Firstly, proliferative lesions are not surgical indications for the left-sided approach, therefore in such cases, the right-sided approach should be considered. Secondly, small tumors in the left thymus (maximum diameter ≤4 cm) can also be safely dissected through right-sided chest surgery, even if the position deviates to the left. Thirdly, cystic lesions, irrespective of size, can be resected by both approaches. However, if the maximum diameter of the solid tumor is more than 4 cm and is inclined to the left, the left-sided approach is recommended.

Pleural adhesion and blockage is recognized as a relative contraindication for thoracoscopic surgery in patients with lung diseases, but unlike lung lesions, mediastinal lesions can be treated using either the left- or right-sided approach [16]. Thus, if the left chest cavity is closed, the right approach is preferred, and vice versa. In the present cohort, one patient experienced a recurrence after resecting the thymus in the right chest, one underwent lung surgery on the right side of the chest, and one had a history of right lung tuberculosis. A computed tomography scan was obtained for all patients. Preoperative X-ray imaging found right pleural adhesions in the above-mentioned four cases, for which the left-sided approach was applied. Therefore, we consider right pleural adhesion or history of surgery on the right as an indication for the left-sided approach.

From top to bottom, the anatomical structures behind the left thymus contain the left innominate vein, aortic pulmonic window, and left pericardium. A large thymoma is usually in close contact with the surrounding structures and is difficult to reveal using the right-sided approach. If injuries to the innominate vein or left pericardium occur in right-sided chest surgery, median thoracotomy and splitting the sternum are necessary for timely treatment. In all 52 patients who underwent left-sided chest surgery, 10 cases were found with tumor invasion into the organs behind the thymus (seven cases involving the pericardium and three involving the left innominate vein). We excised part of the pericardium and sutured it under a thoracoscope in five cases and the other five received left intercostal thoracotomies, which could not have been achieved by the right-sided approach. Therefore, if the left pericardium, left innominate vein, or aortic pulmonic window is involved, the left-sided approach is recommended.

### Considerations for surgery

Surgeons should consider the several steps when deciding upon left-sided chest surgery. First, the design of incision should be considered. As the heart is slightly situated in the left side of the chest, the observation hole should be deviated to the right to avoid interference from the heart, but it should not exceed the midaxillary line, especially for myasthenia patients, otherwise it is impossible to explore the right costophrenic angle through the anterior pericardial retrosternal space (Figure 2). Then, the left mediastinal pleura must be fully opened, especially the superior mediastinal pleura, to expose the innominate vein. During the operation, it is necessary to keep the right mediastinal pleura as intact as possible to prevent herniation to the mediastinum during right lung ventilation, which will affect the surgery. If accompanied by myasthenia symptoms, it is necessary to cleanse the fatty tissue in the right cardiophrenic angle and the surgeon should open the right mediastinal pleura to confirm thorough fat removal. The excision of the thymus is required, followed by the opening of the right mediastinal pleura, and finally the removal of the fat (Figure 5).Left-sided chest surgery is often performed for large tumors, therefore during the operation, the tumor should be removed first and then the thymus should be resected. It is more difficult to expose the left innominate vein than the right vein due to its lack of indication for the superior vena cava. Nonetheless, the left internal thoracic vessel is regarded as an anatomical marker because it is almost parallel to the left innominate vein. Therefore, the mediastinal pleura must be opened next, beneath the internal thoracic vein, and then the thymic tissue should be bluntly dissected from the anterior phrenic nerve to the end to expose the innominate vein. Then the thymic veins must be cut, which can be clearly seen after the bilateral upper thymic lobe is dissected and after clamping them with the titanium clips (Figure 4).The fatty tissue must be removed from the aortic pulmonic window if it is closely connected with the thymic lesions, but the phrenic nerve, pericardial diaphragmatic vessels, and the recurrent laryngeal nerve at the lower edge of the aortic arch must be protected. If the lesion is closely linked to the pericardium, the pericardium must be opened 1 cm beneath the lesion and in front of the phrenic nerve in order to probe the affected area and myocardial involvement under a thoracoscope. If the pericardium is largely involved, it should be removed. If the defect diameter is more than 5 cm and can possibly result in a heart hernia, or if the tumor is invading the myocardium, a thoracotomy is necessary to partly resect the myocardium and repair the pericardium with artificial fabric (Figure 6). If the diameter of the pericardium defect is less than 5 cm, the defect can be repaired with number 1 absorbable sutures using continuous cross-stitches and mesh under a thoracoscope.

## Conclusions

The left-sided approach video-assisted thoracoscopic thymectomy is a safe procedure. Performing a thymectomy using the left-sided approach is more technically demanding than the right-sided approach and it cannot completely replace right-sided chest surgery. It can, however, be a useful alternative procedure to the right-sided approach. Therefore, the surgeon should not only master this operative procedure, but also select its indications accordingly.
